# Why did middle-aged and older people retire since the first COVID-19 lockdown? A qualitative study of participants from the Health and Employment After Fifty study

**DOI:** 10.1186/s12889-023-17548-w

**Published:** 2024-01-05

**Authors:** Stefania D’Angelo, Ilse Bloom, Georgia Ntani, Karen Walker-Bone

**Affiliations:** 1 MRC Lifecourse Epidemiology Centre, University of Southampton, Southampton, UK; 2 MRC Versus Arthritis Centre for Musculoskeletal Health and Work, MRC Lifecourse Epidemiology Centre, University of Southampton, Southampton, UK; 3 NIHR Southampton Biomedical Research Centre, University of Southampton and University Hospital Southampton NHS Foundation Trust, Southampton, UK; 4 Monash Centre for Occupational and Environmental Health, Monash University, Melbourne, Australia

**Keywords:** Retirement, Covid-19 pandemic, Ageing, Qualitative study, Thematic analysis

## Abstract

**Background:**

Governments of Western countries need people to work to older ages, however the COVID-19 pandemic impacted the workforce by pushing older adults to retire. Socio-demographic factors that influence the decision to retire in the pre-pandemic period were, poor or good health, finances, marital status, and gender. The aim of this study was to explore aspects that contributed to the decision to retire among middle-aged and older people in England who retired during the COVID-19 pandemic.

**Methods:**

In September 2022 semi-structured interviews were conducted with a sample of participants from the Health and Employment After Fifty (HEAF) study who retired since March 2020. Consenting participants were purposively selected to achieve a wide spread of characteristics deemed important in the retirement process. Telephone interviews were conducted, audio-recorded, transcribed and then thematically analysed.

**Results:**

24 interviews were conducted (10 men and 14 women, mean age 65 years). Six themes were identified: four of them were non-COVID-19 aspects while two can be interpreted as impact of COVID-19 on the workforce. Work-related factors were of major importance. A sense of appreciation and attachment in relation to their employer, and conversely high work demands and stress, as well as changes in work responsibilities and work practices since lockdown and/or perception of personal safety in the workplace during the pandemic influenced their retirement decision, as did physical and mental health issues. Another theme suggested that some participants felt they had reached the ‘right’ age and needed to spend more time with family. Having the financial capacity to retire was widely mentioned but was never the main factor.

**Conclusions:**

The decision to retire during the pandemic was multi-factorial although changes to work during lockdown were of great importance. Post-pandemic, our findings suggest that there are modifiable aspects of work, including appreciation and fair pay and work conditions, that employers and policy makers could encourage to retain their older workers.

**Supplementary Information:**

The online version contains supplementary material available at 10.1186/s12889-023-17548-w.

## Introduction

The population of Western countries is ageing due to a combination of increase in life expectancy and lower birth rates. Recent UK data published by the Office for National Statistics show that the old-age dependency ratio (the number of people of pensionable age for every 1,000 people of working age) is projected to increase from 280 in mid-2020 to 298 in mid-2030 [1]. To tackle these demographic changes, the UK Government, like many other Governments in the developed world, have implemented policies to encourage people to remain economically active to older ages [2]. In the UK, most people are eligible for state pension, which depends on contributions through the National insurance scheme (taken from pay). The age at which people become eligible for state pension increased in the UK since 2010. Age of eligibility for state pension was 65 years for men and 60 years for women until then and have since undergone a gradual increase dependant on the persons’ sex and date of birth. At the time of the present study, state pension age was 66 years, for both men and women and by 2046, nobody will be eligible for state pension prior to the age of 68 years [3]. Additionally, employers used to be able to force workers to retire at the age of 65 while this is no longer possible since this law was scrapped in April 2011 [4].

Recent analysis of data from the UK Labour Force Survey data shows that contrary to the sustained trend of the previous 10 years, there has been an increase in economic inactivity since the start of the COVID-19 pandemic, particularly in the age group 50 + years, amongst whom the principal reason for economic inactivity is retirement [5]. If sustained, this may suggest that the pandemic will attenuate the effects of the previously successful policy initiatives aiming to keep people in work to older ages [6]. Furthermore, the importance of work, and particularly good quality work, for retaining good physical and psychological health has been previously highlighted [7, 8]. Thus, it is critical to unpick which reasons have prompted middle-aged people to retire since March 2020 to understand whether this trend could be reversed, and people could be motivated to return to work in the post-pandemic era. Equally important for informing strategies to address the challenges would be to understand which reasons have made people delay their retirement despite being close to the typical retirement age.

It is recognised that both poor and good health affect age of retirement: people in poor health are more likely to be forced into early retirement [9–12]. Similarly, individuals in good health are also more likely to take early retirement, wanting to enjoy life while their health allows [11]. Interestingly however, people in good health are also more likely to work beyond state pension age [13]. The association between gender and early retirement is less clear. In a study of Danish employees, women were slightly more likely to take early retirement than men. A possible explanation for this is that women may take on more obligations towards people in their personal situations than men do (e.g., supporting and caring for an ageing parent) [14]. At the same time men are more likely to work beyond the state pension age [13]. Being single, as compared to living with a partner, may also affect the timing of retirement although the evidence is mixed. One study found that retirement was not just a personal decision, but a household decision, especially among married men [15]. Retirement timing also differs depending on occupation and sector, with evidence showing that workers employed in the industry or financial sector tend to retire earlier than those in the service sector [16].

Although factors associated with early retirement are well known in a pre-pandemic context, to our knowledge, they are yet to be explored in the extraordinary circumstances of the global COVID-19 pandemic. Therefore, the aim of this study was to explore aspects that affected the decision to retire since March 2020 in a cohort of older workers in the UK.

## Materials and methods

### Participants and sampling

Participants for this study were drawn from the ongoing Health and Employment After Fifty (HEAF) study, a cohort of over 8,000 men and women recruited in 2013-14 when aged 50–64 years. The original cohort study was set up to assess benefits and risks of remaining in work to older ages as well as to assess the impact of health on employment outcomes. Details of the study design and methodology have been previously described [17]. For the current study we adopted a qualitative design, chosen to provide a deeper understanding of the reasons for retirement in the context of the COVID-19 pandemic. We selected participants who: had responded to the two COVID-19 focussed online surveys (February 2021 and October 2021); were still in paid employment before the start of the pandemic (February 2020); and had reported that they retired at any point since March 2020 (indicated by the response “I decided to retire” to the following question: “Did your employment status change after the start of the COVID-19 pandemic (March 2020) compared to what it was before (February 2020)?”). Eligible participants were sent an email with an introduction to the sub-study, a participant information sheet, and a link to a Qualtrics page where participants were asked to record their written consent and their mobile number. Once written consent was received, we contacted them to arrange for a suitable time for the interview. The sample was chosen to include a wide range of characteristics deemed important in the retirement process and, among consenting participants, we purposively selected those who had some under-represented traits (i.e., living alone, poor health, low financial status). There was no reward for taking part.

### Data collection procedure

Data were collected with semi-structured interviews conducted over the telephone in September 2022. Telephone interviews have been shown to be a good alternative to face-to-face ones [18], and in our case were the most practical option due to the geographical dispersion of participants. A topic guide was developed in advance (See Additional file 1) and used to guide the interviews. Questions were not fixed, and their order was flexible, depending on how the discussion developed with each participant. The interview guide was designed to build trust and rapport with the participant as advised by Barbour et al. [18] and started with some general questions about timing of retirement and characteristics of the job left. Once the interviewee was usually more at ease, we introduced questions about reason/s for retirement and asked whether the pandemic had changed their retirement plans. Finally, we ended with a wrap-up question to give them the chance to add anything we hadn’t covered yet. A series of prompts was available to be used to encourage further dialogue where needed. The topic guide was piloted with IB, an experienced qualitative researcher, as well as with another member of staff who had recently undergone the retirement process herself and was of a similar age of the participants. Interviews were conducted by SD, a female in her late thirties, who introduced herself as a PhD student and researcher in the HEAF study team. Interviews were continued until saturation of themes was reached (i.e. no new codes or themes were identified in the data) [19]. Interviews were audio-recorded (with participants’ consent) and transcribed as soon as possible after taking place.

## Data processing and analysis

We analysed data thematically following guidance by Braun and Clarke [20], and adopted a critical realist position [21], combining a realist approach to ontology and a subjective approach to epistemology. This assumes that there is a real world that we can attempt to understand, however accepting that our knowledge of reality is influenced by the researchers’ background and beliefs [22]. Coding started in parallel to data collection, to be able to monitor for data saturation. Initial codes were identified based on all the transcripts by SD, while double coding was also performed (by SD and IB) for a random sample of data. As soon as complete coding was finalised, candidate themes were derived from the data by grouping codes with similar meaning in an iterative process. We started developing a coding frame by assigning a code name to each section of the transcript; this was accompanied by a description of the code and example quotes. The candidate themes and coding frame were then used to code all the data and then tested by double coding of two random transcripts carried out by SD and IB. Any disagreement was discussed and resolved. We approached this study without a pre-existing conceptual framework, and used an inductive/data driven coding method, meaning that themes did not need to fit into a pre-specified structure [20]. Semantic coding was performed, which meant we did not examine beyond what the participants said. We used Microsoft Office Word© to manage the data analysis process.

## Results

To better contextualise the study, a detailed description of the different phases of COVID-19 lockdown in the UK and what they entailed is presented in the supplementary material (See Additional file 2).

In total, 118 participants were eligible to take part and were therefore invited for interviews. A total of 52 (43%) consented to take part. Because of the nature of the study, we were unable to interview all of them and purposively selected participants from different levels of characteristics potentially important to the retirement process, among those who agreed to take part. A total of 26 interviews were conducted, however only 24 were included in the analysis, meeting the inclusion criteria of having retired since March 2020. Characteristics of participants included (as reported at the latest survey in October 2021) are shown in Table 1. Age at the time of the interview ranged between 59 and 74 years, mean age was 65 years (SD = 3.5 years), 14 were women, and there was a good spread of timing of retirement, with half of the participants in the sample retiring in the period of March-October 2020, and the remaining half retiring after October 2020. Participants of the study were from different categories of ability to manage financially, self-rated health and living arrangements. The interviews lasted between 15 and 30 min, excluding initial introduction and post interview conversations.

**Table 1:** Characteristics of participants included in the interviews

Characteristics	N
**Age (years), mean (SD)**	65 (3.5)
**Sex**	
Male	10
Female	14
**Timing of retirement**	
March - October 2020	11
November 2020 - September 2021	13
**Living arrangements**	
Married/living with partner	18
Living alone	6
**Financial status**	
Comfortable	12
Doing alright	7
Just about managing or struggling	5
**Self-rated health**	
At least good	21
Fair/poor	3

**Fig. 1:**
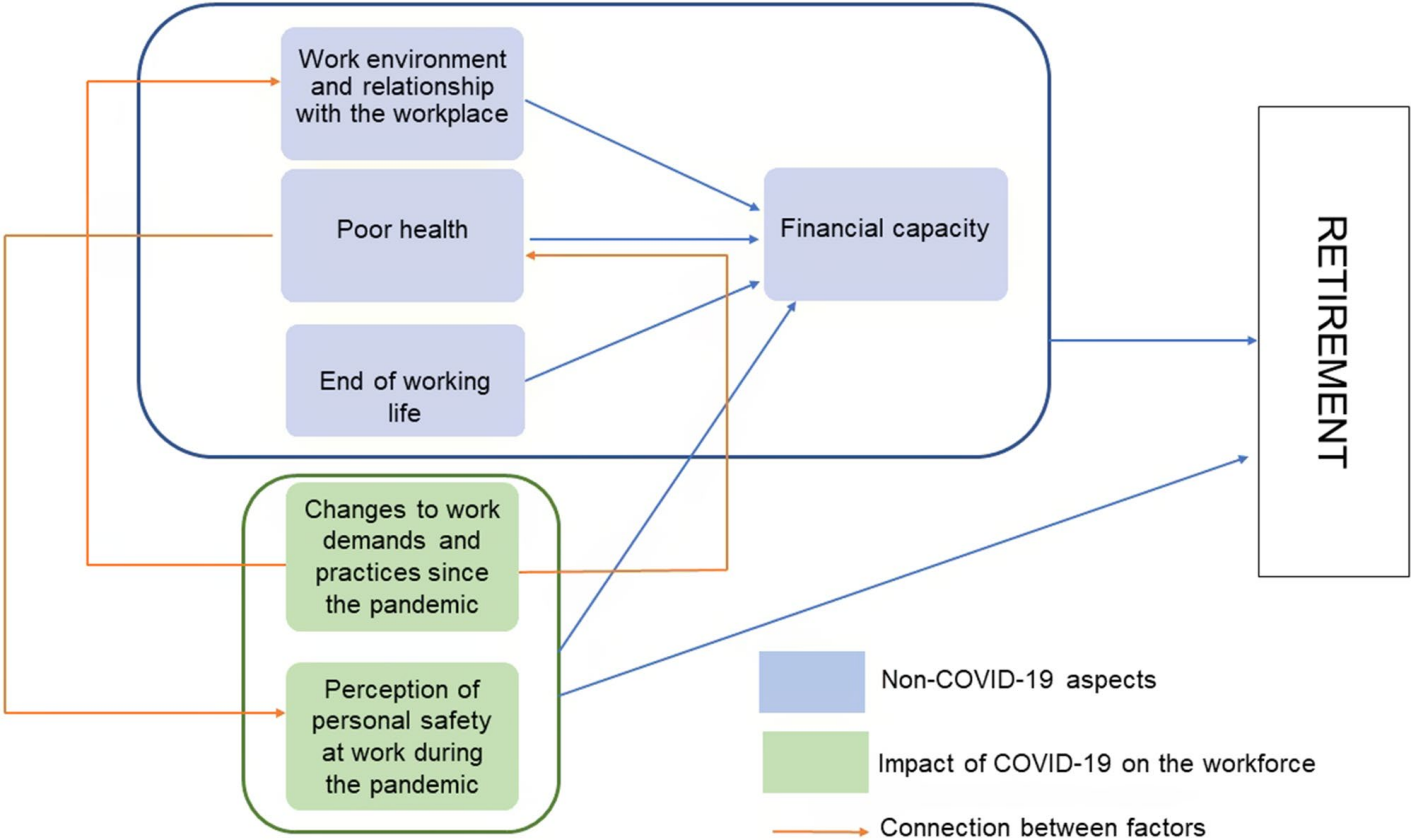
Thematic map for factors that affected the decision to retire. Each theme is described below, with example quotes to illustrate it. Participants’ real names have been replaced with fictitious ones

Six overarching themes were identified as being part of the retirement decision process, and their association with one another is shown in the thematic map of Fig. 1. These themes were: (1) Work environment and relationship with the workplace, (2) Poor health, (3) End of working life, (4) Financial capacity, (5) Changes to work demands and practices since the pandemic, (6) Perception of personal safety at work during the pandemic. The first four summarise non-COVID-19 related themes, while the remaining two related to the impact of COVID-19 on the workforce. Both domains tended to interact extensively in dictating the timing of retirement.

### Work environment and relationship with the workplace

This theme captures aspects of the work environment that were already in place before the pandemic started and that played an important role in the decision to retire.

In most cases, the work environment acted in combination with other elements to result in retirement. Participants reported work stress and long working hours as important reasons for their decision:

*‘There was a lot of stress with it [work], trying to keep up with everything. There used to be two people in where I used to work and then they took one person away and I had to do everything by myself… so that yeah started stressing me out and that’s when I turned around and said when the pandemic comes that’s it bom finito’.* Phil, 67 years.

While for others the problem was lack of staff and high work demands and pressure:

*‘…they [manager] were also expecting more out of everyone. I have been there quite a few years, when I first started… there were 40–50 people working in that store… now they are running the whole store on 15 people… But they would constantly try to get more out of you and if you weren’t willing to work when they wanted you, you’ve got your ordinary hours but if you also weren’t willing to work you know, extra here, there, and everywhere, then you weren’t one of the team. Attitudes were squeezing you’.* Iris, 61 years.

Others praised their employers for accommodating their individual needs and for being flexible. Participants reported that a feeling of attachment and appreciation towards the employer meant that they would have felt guilty leaving the job during lockdown, when the employer needed people with long expertise:

*‘I didn’t feel I could leave my company at a time like that, with the experience I’ve got over the years and I’ve been treated very well by my company, so morally I didn’t think it was right at the time’.* Matt, 64 years.

### Poor health

This theme emerged from participants who reported some pre-existing health problems such as hip arthritis, painful menopause, anxiety, or depression which played a role in their decision to retire. Often these conditions interacted with pandemic- associated changes at work, increasing the participants’ concerns about their risk of catching the virus in the workplace and therefore leading to their decision to retire. In some cases, participants retired because their job was no longer compatible with existing health issues:

*‘I retired because I couldn’t do the job. Well, I was let go. They went through a process so that I couldn’t do the job. I couldn’t do the job because of my health and so I was dismissed… I started off…I was based quite often mostly in the office and doing a lot of the paperwork and things like that and then things changed, the owners of the company changed, and everyone was expected to go out on the shop floor’.* Iris, 61 years.

Or they reported a recent loss of a family member, and the effect such event had on their health, as the reason why they left their job:

*‘… and when I went back [to work] I knew I really couldn’t cope with looking after palliative care patients so soon after losing husband and my mum but I’d also developed really bad arthritis in my hip and couldn’t cope with in and out’.* Karen, 66 years.

### End of working life

This theme emerged from a group of participants who stated that the decision to retire pre-dated the pandemic, but that the pandemic, lockdown, and/or changes to the workplace during the pandemic appeared to have accelerated the process. Participants reported the need for relaxing after many years of work:

*‘I was coming up to retiring as I had been at work ever since I was 16…not really having time off at all so from job to job to …out and so never really had gap year or anything…so forty odd years, well almost fifty years working so I thought that’s about time I give myself a rest and go out enjoying my motorcycle and that’.* Phil, age 67.

While others reported wanting to spend more time with the family:

*‘yes family as well, you know, my husband was retired and I’ve got grandchildren who I do look after, …. So … my husband being retired as well it was nice then, you know, spend a little bit of time together’.* Julia, age 67.

### Financial capacity

Although their financial status was never reported as the main reason for retiring, participants took the decision to retire only when their finances allowed it. Some participants had carefully planned financially for their retirement for some years:

*‘we planned for them so we had been planning for the ten years so essentially I had a mixture of pensions…so I had…work place pensions which were a final salary scheme with 3 companies……so it was sort of all planned out’.* Tony, 66 years.

While others decided to stop working when they no longer needed to financially support the children:

*‘… But once that [financially supporting the children] was virtually sorted, the need for work actually came to an end so hence you know, the additional reason for actually retiring’.* Leo, 66 years.

### Changes to work demands and practices since the pandemic

This theme reflects what participants reported about changes to work that occurred during lockdown, ranging from changes to work practices and responsibilities, to changes to working patterns (including shifting to home working), changes to workload or work demands. Some participants reported that working from home had its challenges and resulted in increased loneliness, frustration and had a negative impact on their physical and/or mental health, feeding into the poor health theme.

The shift to home working had such a negative impact on Linda’s mental health that her physical health suffered too, and she stated that she felt she had no choice but to retire:

*‘It was probably the end of April when I started to work in the house…I did find extremely difficult working at home. I like to be out and about, I am a very busy, active person and… and I like to be in company, I don’t like being on my own … my husband was still working from his office… so I was basically in the house on my own… and I was always used to go to the gym… I was very active and I put weight on… and I am not a one for going for a walk on my own because I like company, so I didn’t feel like the urge to get out and go for a walk, so I wasn’t really getting any fresh air. So really it was a bit of a downwards period if I am honest. Obviously a bit with my mental health. I mean I’m alright now, but I did find it really hard, luckily the house I live in, you can walk right around the staircase into the four different rooms, so I used to just pace around it (laughs), you know, imagine an animal in a cage, trapped, that’s what it felt like’.* Linda, 64 years.

While others expressed their appreciation of home working which enabled them to keep working. They emphasized that if they had been required to continue working from the office and commuting, they would have retired earlier than they eventually did. They spoke of the good qualities of home working such as its flexibility:

*‘…I enjoyed working at home because you didn’t have to face other people and you were out of the office politics in that case, you know, when you were working at home, so it suited me better really’.*

*‘And I liked not having to get up and get dressed first thing in the morning, you know. I used to start very early and then I’d do a couple of hours work and then I’d sign off for half an hour and get dressed and have some breakfast and things like that, yes I found it more flexible’. Sue, 67 years.*

Some spoke of the lack of commute and not having to face office politics as factors that suited them better, enabling them to keep working. Another participant reported on the importance of having a good workstation set up at home and that had enabled them to feel good about continuing to work.

In other cases, the drastic changes to work practices, or to the day-to-day job during lockdown, made their job no longer enjoyable and consequently motivated them to retire:

*‘so we went into the first lockdown and that’s completely changed the way in which the community rehabilitation service was working and we just stopped going to see patients… and nobody knew about how to go about anything, and nobody knew what we should do and what we shouldn’t do and at the same time as that there was a great deal of discussion about who could and couldn’t be patient facing and my husband had chronic lymphocytic leukaemia so he is obviously clinically vulnerable … and the decision was taken at work that people like me… would not be allowed to see patients until a vaccine came about and that meant that they wanted me to return to desk-based project work and research … I didn’t agree with the complete cessation of input to patients…so I took the decision in the end that if, that I didn’t want to do the work that was being offered to me in leu of clinical work…so I took retirement!’.* Lily, 65 years.

*‘Yes, the main reason was due to COVID, my job actually became very difficult, because the hospice was no longer allowing visitors in, I was no longer able to greet visitors, say goodbye to visitors, help them in any way at all we had to wear full PPE throughout the day so it was 8 hours and because we couldn’t have shared workspace it meant that I couldn’t leave the desk for 8 hours…and it became very difficult, very upsetting. We had a lot of upset visitors stood outside and we couldn’t offer them a cup of tea, there was nothing in my job that I used to do day in day out for hundreds of visitors, and we were restricted to maybe six visitors a day if the patient was actually at the point of dying. So, it became very difficult, very emotional, and very long days doing nothing, and it got to a point where I thought I can’t do this anymore, I have to retire’.* Elaine, 66 years.

### Perception of personal safety at work during the pandemic

In this theme, some participants expressed their anxieties about attending the workplace if they felt that COVID-19 safety procedures were not implemented. This was closely interconnected with the ‘poor health’ theme as such perception of not being safe in the workplace was especially true for participants with comorbidities:

*‘I also had to shield during the pandemic because as part of the Hodgkin’s I had a thymectomy so made me immune-supressed so that made me high risk, I worked in a school… so obviously I felt that was quite a risky thing, I was quite scared to go back in the offices, with all the children and people didn’t really seem to be observing social distancing, and all the staff were handling the same files, it felt too risky’.* Lucy, 59 years.

or those living with a vulnerable person:

*‘Basically… it was a kind of joint decision it might sound a bit selfish but I was thinking my other half decided that because she has got slight…she was at slightly high risk because of asthma she wasn’t happy about me having to drive coaches with school kids around without any masks or anything like that (hmm)’.* Leo, 66 years.

Sophia described the stress of potentially passing on the virus to her students as a factor which she perceived contributed to her decision to retire:

*‘So it was a very tense situation because … the covid thing hanging over your head, so at that point in time there were no vaccinations, so nobody had any protection against getting even covid, and we were more likely to take it into the prison than contract it from the prison, so that was a lot of stress if you like about teaching people on a 1 to 1 basis, when you didn’t really know if you were infecting them’.* Sophia, 65 years.

On the contrary, others reported they never worried about the safety of their workplace:

*‘No, it wasn’t a worry because we have always been extremely clean and more than cautious, you know we’ve been more than cautious for years so every surface and every handle and everything and the windows and doors have been open forever. And every member of staff was in full PPE and nobody was actually allowed in, so it was an extremely clean environment. It was more worrying going to a local shop than it was to actually be at work’.* Elaine, 65 years.

## Discussion

In this qualitative study we identified six themes summarising what UK middle-aged and older people reported about their decision to retire since the beginning of the COVID-19 pandemic. These themes were: work environment and relationship with the workplace, poor health, end of working life, and financial capacity, which were non-COVID-19 aspects, while changes to work demands and practices since the pandemic and perception of personal safety at work during the pandemic, appeared to be themes describing the impact of COVID-19 on work.

Our findings reveal strong interconnection between various themes. Purely COVID-19 aspects that affected the workforce interacted with existing non-COVID-19 domains in the retirement thinking process. There were instances where, if there was financial capacity, changes to work demands and practices that occurred with lockdown adversely affected health and accelerated their retirement process. While for others, such sudden changes to work interacted with their established work environment and relationship with the workplace in determining the timing of retirement. Additionally, some individuals cited poor health as a reason for premature departure from work, meaning their risk perception in the workplace was heightened by their health conditions.

Nevertheless, changes to work demands and practices precipitated by the pandemic, as well as feeling unsafe in the workplace due to lack of strict COVID-19 protocols, were the most important reasons that participants gave for their retirement. However, it is noteworthy that the decision to retire was mostly multi-factorial, as has been consistently reported about retirement in the pre-pandemic period [23, 24].

Most of the aspects which encouraged participants to remain in work for longer were also work-related, such as attachment and gratitude towards the employer, or appreciation of the changes to work routine that occurred since the pandemic (i.e., working from home). Most participants stated that the pandemic and its consequences changed their retirement plans, as was found in The Over 50s Lifestyle study, in which 63% of adults aged 50–70 who left their job during the pandemic, reported that they did so earlier than intended [25]. Another qualitative study of 19 participants who were either already retired or were over 55 years but still employed when interviewed, reported that the pandemic had significantly changed their plans and expectations of retirement [26]. In line with our findings, the effect of the pandemic was not uniform across the sample, with some appreciating the flexibility of working from home while others reporting that the pandemic had brought forward their retirement. Identifying whether factors that affected the decision to retire are potentially modifiable is of great importance at a time when several western countries, including the UK, are experiencing an increase in economic inactivity. A report from the Resolution Foundation [27] has shown that, since the beginning of the pandemic, the UK has seen the biggest fall in work participation compared with other OECD countries (especially in the age group 55–64 years) although work participation in this country remains significantly higher than other OECD countries. One of the main reasons for such an increase in economic inactivity is the rise in people who have taken retirement. Therefore, the Government are currently implementing policies, such as increase in tax relief on pensions [28], to bring back to work some of the early retirees. As well as ‘healthy’ retirement, ill-health retirement has been responsible for part of the recent increase in economic inactivity. In the UK, the number of working-age people who became inactive due to long-term sickness has dramatically risen since the beginning of the pandemic [27]. Findings from the present study suggest that this may be attributed to existing health issues, coupled with participants’ perceptions of not being safe in the workplace because COVID-19 safety procedures were not adequately followed, which together played an important role into their retirement decision. It needs to be acknowledged however, that there were several occasions where participants reported to be in good health and that health had no role whatsoever in their decision to retire. For most people, work is not only a way to fulfil their material needs, but is a key determinant of social engagement, self-esteem, sense of purpose and achievement [7].

Our findings suggest that the pandemic has accelerated the retirement process for some participants who were pushed into retirement prematurely, e.g. due to being in poor health. Overall, the pandemic might have pushed people with poorer financial situations (and in relatively poorer health) to stop working. As work plays an important role in retaining good physical and psychological health [7], if people with poor socio-economic status were forced to stop working during the pandemic this is likely to lead to a further deterioration in their health. Thus, it is concerning that overall, the pandemic might have contributed to widening health inequalities. Further research will be needed to investigate the extent to which people with poorest socio-economic status were forced to stop working during the pandemic.

In our data, one theme (end of working life) appears non-modifiable, as people who follow this retirement pathway had mostly made the decision about retirement pre-pandemic, although some recognised that lockdown might have accelerated their retirement plans. It is noteworthy that this sample of participants retired soon after the new UK regulations on state pension age entitlement came into force, however participants never mentioned that having reached state pension was a reason for retiring. Financial capacity to retire acted as an important contributing factor to retirement, and in most cases, it was necessary to allow participants to make the decision, but most participants implied it was not the triggering factor. One other theme identified however, that of changes to work demands and practices, is likely to be potentially modifiable. The role of changes in work responsibilities, work practices, and day-to-day job since the pandemic was particularly important, and in the wake of the pandemic, employers need to take those into account if they want to retain their workforce. On the contrary, our findings show that factors that motivated people to remain in work were feeling connected with employers and colleagues, having a work management that is understanding and accommodating towards individual’s needs, and job flexibility; the role of those factors have been previously shown [23] to be important to keep people in work to older ages in the pre-pandemic period and need to be accounted for by employers particularly in the wake of the COVID-19 pandemic. A good step in that direction was the recently passed Flexible Working Bill, which entitles employees across the UK to have more flexibility over when and where they work [29].

The pandemic has accelerated changes in the mode of working and, three years on, it is unlikely work will ever return to how it was pre-pandemic. One of the main challenges for the near future appears to be the need for employers to align with employees’ preferences. Almost half of the participants of a recent survey reported that their organisation did not consult them about their preference of work practice, which might translate into a mismatch between their preference and employer’s policy, with the potential of pushing employees to leave the workforce [30].

Retirement transition for this sample occurred at a time when older adults were portrayed unfavourably in the media, often being depicted as a vulnerable group who can offer little to society [31, 32]. Although this aspect was not mentioned in the interviews, it is possible that it was a latent feeling among participants of the study.

Our findings need to be considered alongside some limitations. Although the HEAF study is representative of the wider middle-aged population [17], this sub-sample is likely to be not without bias, as participants who completed the online surveys (and were therefore eligible to be interviewed) were more likely to have better socio-economic status and health compared with those who did not [33]. However, due to the high response rate (43%), we had a large pool of participants to choose from and we could purposively select participants with a wide range of characteristics. Therefore, these interviews still provide a unique and detailed picture of retirement experiences since the first national lockdown across a wide range of socio-economic and health factors. When participants were invited to take part in the interviews, it was explained that the aim of the study was to explore the reason(s) that fed into their decision to retire, therefore it is possible that only participants with strong opinions about the effect of the pandemic on their recent retirement agreed to take part. We acknowledge that these findings are potentially limited to the UK, due to the difference in the implementation of lockdown regulations and to differences in the social insurance systems across different countries. Finally, the changes to the pension system that the UK was undergoing at the time of the study were not considered when we designed the study.

This qualitative study represents the view of this research team and we do not exclude that other researchers might have come up with slightly different themes. Respondent validation to ensure rigor of the findings was not an option in this instance, due to time constraints. However, we adopted double-coding throughout the analysis phase. The research team included people with a range of expertise, who met frequently to discuss findings, resolve any discrepancy that arose during the coding process, and agreed on the coding frame. SD was involved in both the data collection and data analysis and was therefore fully embedded into the whole process. All these aspects increased the reliability and rigor of our findings.

## Conclusions

In summary, middle-aged and older people in England reported a variety of different reasons for retiring since March 2020 (i.e. the start of the first COVID-19 pandemic lockdown). Work-factors contributed importantly amongst this group of participants from the HEAF study. This qualitative study has shown that sudden changes to work arrangements and day-to-day work were mostly not appreciated by older workers, while feeling connected with employers and colleagues, having a work management that is understanding and accommodating towards individual’s needs, and job flexibility motivated people to delay their retirement. These potentially modifiable work factors could be considered by employers and the UK Government if the aim is to retain the older workforce and bring recent retirees back to work.

### Electronic supplementary material

Below is the link to the electronic supplementary material.

Supplementary Material 1

Supplementary Material 2

Supplementary Material 3

## Data Availability

The data are not freely available owing to data protection and consent restrictions, as data contain sensitive and potentially identifying participant information even after de-identification. The data may be accessed by collaboration with the HEAF study team. Details on how to get in touch with the study team are available at: https://generic.wordpress.soton.ac.uk/heaf/contact/. Queries should be addressed to Ms Stefania D’Angelo (corresponding author).

## Abbreviations

HEAF: Health and Employment After Fifty study
OECD: The organisation for economic cooperation and development
