# Supplementary material for: Why did middle-aged and older people retire since the first COVID-19 lockdown? A qualitative study of participants from the Health and Employment After Fifty study
Source: BMC Public Health. 2024 Jan 5;24:103. doi: 10.1186/s12889-023-17548-w (PMC10770915; doi:10.1186/s12889-023-17548-w)
Supplement: Supplementary file 1 — Supplementary Material 1 [file 12889_2023_17548_MOESM1_ESM.docx]

# Phases of lockdown in the UK

On the 23rd of March 2020 the British prime minister announced the beginning of the first lockdown: people had to remain at home expect for shopping for basic necessities, medical needs and performing one form of exercise per day. People were urged to work from home whenever possible, except for essential workers. In June 2020 a phased reopening of society started with re-opening of schools, followed by re-opening of businesses such as pubs, restaurants, and hairdressers in July 2020. In August 2020, lockdown restrictions eased further with the re-opening of indoors theatres and, at the same time, sheltering of clinically vulnerable people officially ended. In November 2020, England entered its second national lockdown which lasted four weeks. On the 6th of January 2021, England entered its third national lockdown which started to ease in March 2021 with the re-opening of schools. In July 2021 most legal limits on social gatherings were removed in England and the remaining closed sectors finally reopened.

The rollout of COVID-19 vaccination started in December 2020 and aimed at reaching the greatest number of people at risk, in the shortest possible time. People who were prioritised then were residents of care homes and their carers, people over the age of 50, those clinically extremely vulnerable, and health and care workers. In April 2021, the rollout was extended to people in the younger age groups. Finally, from August 2021, 16- and 17 year-olds were also invited for a single dose of the vaccine.
