# Supplementary material for: Why did middle-aged and older people retire since the first COVID-19 lockdown? A qualitative study of participants from the Health and Employment After Fifty study
Source: BMC Public Health. 2024 Jan 5;24:103. doi: 10.1186/s12889-023-17548-w (PMC10770915; doi:10.1186/s12889-023-17548-w)
Supplement: Supplementary file 2 — Supplementary Material 2 [file 12889_2023_17548_MOESM2_ESM.docx]

## HEAF COVID-19 Qualitative study - Interview guide

**Timing of retirement and characteristics of job left**

- Please can you tell me when (approximately) you retired?
- *What was your job at the time you retired? What did it involve? How many hours were you working?*

**Experience of retirement**

- How are you finding retirement? Is life in retirement as you had anticipated? [expand by asking them in which way] What do you think about retirement life at this stage? [Are you satisfied with your decision of retiring?]
- What are your thoughts about going back to work in any kind of form in the foreseeable future?
- How did COVID-19 affect yourself and your family, apart from your work? Were you afraid of catching covid?

**Reasons for retirement**

- Now, please think about the reason/s that were responsible for your decision to retire. I appreciate there might be a combination of reasons behind this decision. Could you tell me what you believe was the main factor?
- What were any other reasons that led you to retirement?
- *What made the decision to retire more difficult? [Depending on whether this is mentioned above or not] What were your retirement plans before the pandemic. How did they change since lockdowns? [Would you have retired at the same time if COVID-19 had not occurred] [if applicable: What caused this change to happen?]*
- What, if anything, might have encouraged you to work for longer than you did?

**Wrap-up question**

- Is there anything we haven’t mentioned yet which you would like to discuss?
